# Compartment-Specific Proximity Ligation Expands the Toolbox to Assess the Interactome of the Long Non-Coding RNA NEAT1

**DOI:** 10.3390/ijms23084432

**Published:** 2022-04-17

**Authors:** Victoria Mamontova, Barbara Trifault, Kaspar Burger

**Affiliations:** 1 Mildred Scheel Early Career Center for Cancer Research (Mildred-Scheel-Nachwuchszentrum, MSNZ), University Hospital Würzburg, Josef-Schneider Str. 2, 97080 Würzburg, Germany; victoria.mamontova@uni-wuerzburg.de (V.M.); barbara.trifault@uni-wuerzburg.de (B.T.); 2 Department of Biochemistry and Molecular Biology, Biocenter of the University of Würzburg, Am Hubland, 97074 Würzburg, Germany

**Keywords:** proximity ligation, paraspeckles, NEAT1, long non-coding RNA, cancer

## Abstract

The nuclear paraspeckle assembly transcript 1 (NEAT1) locus encodes two long non-coding (lnc)RNA isoforms that are upregulated in many tumours and dynamically expressed in response to stress. NEAT1 transcripts form ribonucleoprotein complexes with numerous RNA-binding proteins (RBPs) to assemble paraspeckles and modulate the localisation and activity of gene regulatory enzymes as well as a subset of messenger (m)RNA transcripts. The investigation of the dynamic composition of NEAT1-associated proteins and mRNAs is critical to understand the function of NEAT1. Interestingly, a growing number of biochemical and genetic tools to assess NEAT1 interactomes has been reported. Here, we discuss the Hybridisation Proximity (HyPro) labeling technique in the context of NEAT1. HyPro labeling is a recently developed method to detect spatially ordered interactions of RNA-containing nuclear compartments in cultured human cells. After introducing NEAT1 and paraspeckles, we describe the advantages of the HyPro technology in the context of other methods to study RNA interactomes, and review the key findings in mapping NEAT1-associated RNA transcripts and protein binding partners. We further discuss the limitations and potential improvements of HyPro labeling, and conclude by delineating its applicability in paraspeckles-related cancer research.

## 1. Introduction

RNA metabolism is a central component of gene expression. During the life cycle of a transcript essentially all RNA molecules form secondary structures and associate with RNA-binding proteins (RBPs). RBPs not only facilitate the folding, processing and maturation of transcripts, but are also critical for the localisation, stability and functions of many RNA transcripts. The human genome contains approximately 19,000 protein-coding genes that are differentially expressed in individual cells and developing tissues [1]. However, only about 1.5% of the total RNA amount accounts for messenger (m)RNA. On the other hand, the vast majority of the transcriptome is synthetised from a diverse array of non-coding RNA loci. All three RNA polymerases produce non-coding transcripts, which are grouped into two classes, primarily based on their length. The class of small non-coding (snc)RNA contains transcripts that typically are shorter than 200 nucleotides such as micro (mi)RNA, enhancer (e)RNA, transfer (t)RNA, and small nuclear (sn)RNA. Longer transcripts such as ribosomal (r)RNA, intronic as well as intergenic transcripts and promoter-associated antisense transcripts belong to the class of long non-coding (lnc)RNA [2,3]. In the human genome, a staggering number of 60,000 non-coding loci were gleaned from technological advances in RNA sequencing and bioinformatics, which includes more than 10,000 lncRNA loci [4,5,6]. The high number of lncRNA loci reflects their widespread roles, both as functional regulators of gene expression [7,8] and as structural organisers of subcellular compartments in mammalian cells [9,10,11,12,13,14]. The bulk of lncRNA lack typical features of protein-coding transcripts such as the requirement for pre-mRNA splicing and polyadenylation for maturation. In addition, lncRNA are often expressed at low levels and the biochemical and genetic tools to tackle them are somewhat limited. Thus, technological barriers often challenge the enrichment of lncRNA and the analysis of associated RBPs, which is a prerequisite to investigate lncRNA functions. 

Interestingly, a flurry of recently developed methods to isolate lncRNA and identify their associated transcripts and proteins allows querying of lncRNA interactomes with increasing selectivity and specificity [15,16]. In this review, we discuss the Hybridisation Proximity (HyPro) labeling technology, a novel method to map RNA interactomes by compartment-specific proximity ligation in the context of other methods to study RNA interactomes. Unlike many other existing methods to study the interactome of disease-associated transcripts, HyPro labeling promises to be of great interest for cancer research, as it requires a relatively low amount of starting material and is applicable to primary and patient-derived cells. The HyPro technology has recently been applied to identify the transcripts and proteins that are associated with three lncRNA transcripts, which seed the nucleolus, nuclear paraspeckles and the perinucleolar compartment (PNC), respectively [17]. With focus on paraspeckles and the nuclear paraspeckle assembly transcript 1 (NEAT1) as a paradigmatic lncRNA target, we describe the principles of the HyPro labeling method and discuss the key findings, strengths and limitations of the technology. We further delineate the applicability of HyPro labeling as a potentially powerful tool to investigate the molecular principles that contribute to the deregulation of NEAT1 transcripts in cancer and promote NEAT1-dependent tumourigenesis.

## 2. NEAT1 Isoforms Modulate Gene Expression

**Figure 1:**
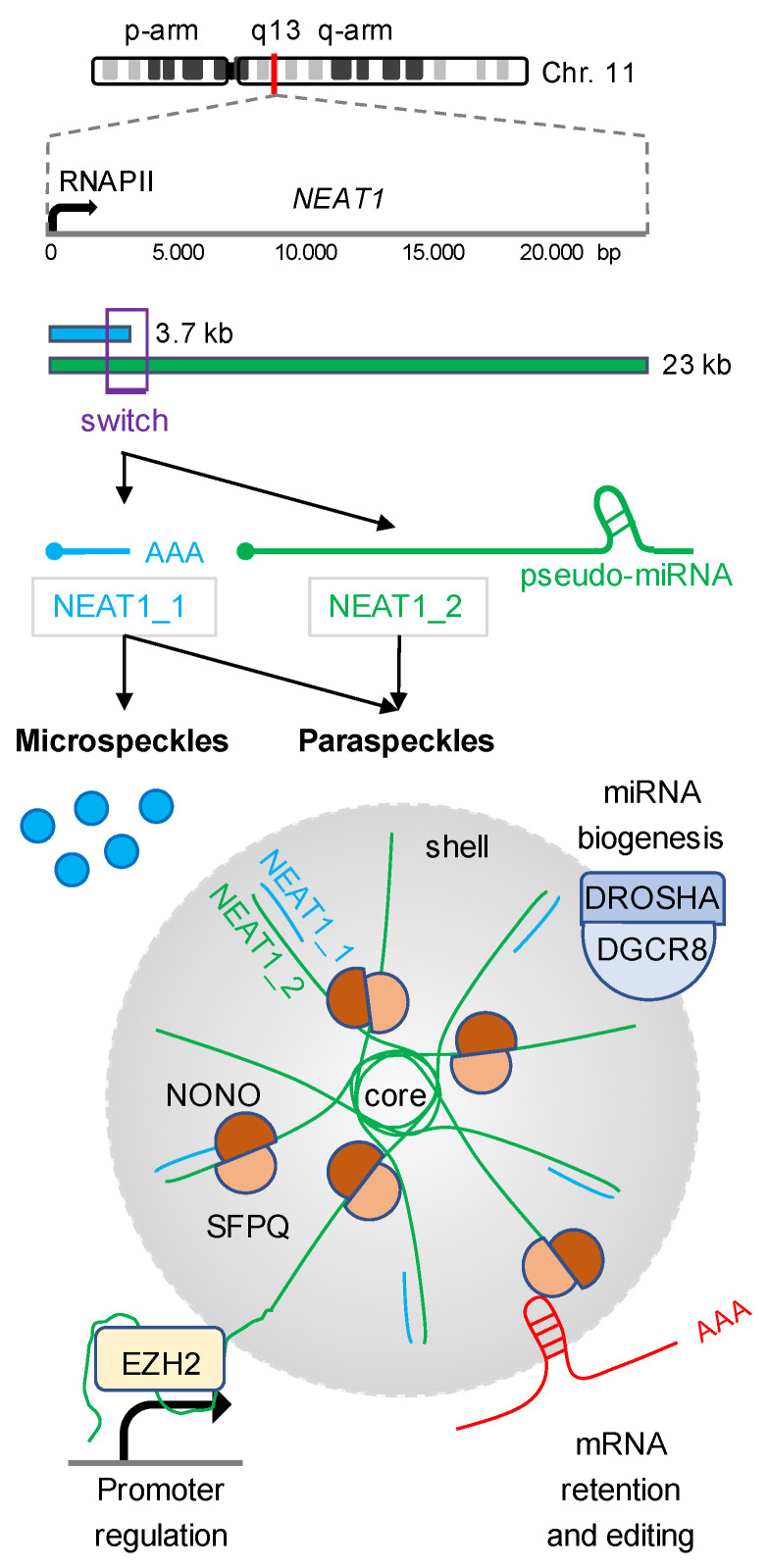
Two NEAT1 isoforms modulate gene expression at the transcriptional and posttranscriptional level. The human NEAT1 gene is located on chromosome 11q13 (red line) and encodes two overlapping isoforms, NEAT1_1 (blue) and NEAT1_2 (green), which are both produced by RNAPII transcription. NEAT1_1 synthesis requires cleavage and polyadenylation (AAA) at the size of 3.7 kb (isoform switch region, purple box), whereas NEAT1_2 is 23 kb long and a of suppressed polyadenylation and contains a pseudo-miRNA hairpin and a triple helix structure at the 3′ end. NEAT1_1 is present in both microspeckles (blue) and paraspeckles (grey); both not essential. NEAT1_2 is the essential structural component of paraspeckles, which are membrane-less nuclear condensates that comprise a core-shell arrangement and form via phase separation. Paraspeckles contain more than 40 RNA binding proteins such as NONO and SFPQ, and modulate the RNA metabolism by three major mechanisms: (i) the recruitment of the DROSHA/DGCR8 microprocessor complex (purple) to stimulate miRNA biogenesis, (ii) the retention and editing of a subset of mRNAs (red), and (iii) the modulation of protein-coding gene promoters (black arrowhead) by scaffolding chromatin modifying enzymes such as EZH2.

Paraspeckles are phase-separated nuclear bodies that are built on the lncRNA NEAT1 and regulate gene expression and the RNA metabolism in the interchromatin space of mammalian nuclei [18,19,20,21,22]. The NEAT1 locus encodes two GC-rich, RNA polymerase II (RNAPII)-dependent isoforms: NEAT1_1 and NEAT1_2 (Figure 1). The expression levels of NEAT1 and the ratio of the two isoforms are dynamic and regulated at various levels. During development, for instance, the protein arginine methyltransferase 4 (PRMT4/CARM1) inhibits RNAPII transcription at the NEAT1 locus by direct methylation of the RNAPII carboxy-terminal domain (CTD), which impacts on the lineage choice in the developing embryo [23,24,25]. The synthesis of NEAT1 isoforms is further regulated by alternative 3′ end processing. The maturation of the nascent NEAT1_2 transcript is promoted by the heterogenous nuclear ribonucleoprotein K (HNRNPK). HNRNPK is an essential paraspeckles component and inhibits the binding of the cleavage and polyadenylation specificity factors 5 and 6 (NUDT21, CPSF6) to the isoform switch region of the nascent NEAT1 transcript. HNRNPK impairs premature termination of RNAPII transcription and promotes the formation of non-polyadenylated, full length NEAT1_2 transcripts in human tissue culture cells [26]. On the other hand, many components of the multimeric Integrator complex, including the catalytically active Integrator complex subunit 11 (INTS11), are also enriched in paraspeckles. The Integrator is involved in the 3′ end formation of a subset of sncRNA transcripts such as snRNA and eRNA [27,28,29], and promotes the synthesis of NEAT1_1 by stimulating cleavage and polyadenylation of nascent NEAT1 transcripts within the isoform switch region in HeLa cells [30]. Likewise, the paraspeckle component TAR DNA-binding protein 43 (TARDBP/TDP-43) enhances NEAT1_1 polyadenylation in pluripotent cells [31]. The expression levels of NEAT1 transcripts are also controlled at the epitranscriptomic level. Four bona fide N^6^-methyladenosine (m6A) modification sites have been mapped in close proximity to the 5′ end of NEAT1 transcripts [32]. The m6A marks are placed by the RNA methyltransferase 3 (METTL3) and stabilise NEAT1 transcripts in various cancer cells, including renal cell carcinoma and chronic myeloid leukaemia [33,34].

### 2.1. Posttranscriptional Gene Regulation by Paraspeckles-Associated NEAT1

Interestingly, the two NEAT1 isoforms have both separate and partially overlapping functions in mammalian cells. The 23 kb long NEAT1_2 isoform is the structural component of nuclear paraspeckles and functions as a multipurpose molecular scaffold that modulates the shape of paraspeckles and the association of RBPs and transcripts with paraspeckles [35,36]. NEAT1_2 undergoes a core-shell arrangement and sequesters more than 40 RBPs. The non-POU domain containing octamere-binding protein (NONO) and the splicing factor proline and glutamine rich (SFPQ), for example, are core components of paraspeckles and are required for the retention and editing of a subset of pre-mRNA transcripts [37,38,39]. A number of transcription factors and RBPs related to neurodegenerative diseases, most prominently amyotrophic lateral sclerosis (e.g., FUS; EWSR1; TARDBP; TAF15), and the cellular stress response (e.g., CIRBP; UBAP2L), are associated with NEAT1_2 in paraspeckles as well [40,41,42]. Paraspeckles also function as a hub for miRNA biogenesis. NEAT1_2 contains a pseudo-miRNA near its 3′ end, which serves as a bait for RNAi factors and allows efficient miRNA processing by sequestering the DROSHA/DiGeorge syndrome critical region 8 (DGCR8) microprocessor complex to the shell region of paraspeckles in HeLa cells [43]. Indeed, numerous studies link NEAT1 to miRNA functions and report NEAT1- and miRNA-dependent modulation of signaling pathways, which are reviewed elsewhere [44,45]. 

### 2.2. Posttranscriptional Gene Regulation by Cytoplasmic NEAT1

The 3.7 kb short NEAT1_1 isoform is less described. NEAT1_1 is also found as a component of paraspeckles, but seems to be non-essential for paraspeckle formation. Instead, NEAT1_1 accumulates in microspeckles, which are small spot-like nuclear bodies that may function independent of paraspeckles in U2OS cells [46]. Intriguingly, novel functions for the NEAT1_1 isoform have recently been described in the cytoplasm. NEAT1_1 is overrepresented in breast cancer cells and promotes the metabolism of glucose to stimulate growth and metastasis [47]. Upon nuclear export by the RBP Pinin, NEAT1_1 scaffolds the assembly of a ribonucleoprotein complex that contains the three glycolytic enzymes phosphoglycerate kinase 1 (PGK1), phosphoglycerate mutase 1 (PGAM1) and enolase 1 (ENO1). The complex enhances the penultimate step in glycolysis, which sustains high proliferation and drives tumourigenesis independent of nuclear paraspeckles. Cytoplasmic NEAT1_1 has also been described as a regulator of stem cell self-renewal in acute myeloid leukaemia (AML) [48]. In contrast to its role in breast cancer, however, NEAT1_1 functions as a tumour suppressor in non-transformed blood cells. NEAT1_1 suppresses leukemogenesis and leukemic stem cell function through interaction with the cytoplasmic dishevelled segment polarity protein 2 (DVL2) and the E3 ubiquitin ligase tripartite motif containing 56 (TRIM56/RNF109), which stimulates the degradation of TRIM56 and prevents the onset of cancerogenic Wnt signaling. Consequently, NEAT1_1 is downregulated in AML.

### 2.3. Cotranscriptional Gene Regulation by Chromatin-Associated NEAT1

Recent work suggests that NEAT1 transcripts are also involved in the regulation of chromatin accessibility. The depletion of NEAT1 in colorectal cancer cells, for example, leads to widespread loss of histone H3 lysine-27 acetylation, a histone mark associated with open promoters and active transcription, suggesting that the presence of NEAT1 on chromatin may also be required for open chromatin and the organisation of higher-order chromatin structure [49]. Indeed, NEAT1 transcripts accumulate on several hundred protein-coding gene promoters and scaffold the recruitment of histone modifying enzymes, chromatin modellers and transcription factors such as the enhancer of zeste 2 poycomb repressive complex subunit 2 (EZH2) and the WD repeat domain 5 protein (WDR5) to modulate RNAPII transcriptional activity in human tissue culture cells [50,51,52,53,54,55,56]. Intriguingly, NEAT1 may also directly transactivate RNAPII. NEAT1 serves as a bridge to facilitate the binding between the major regulatory transcriptional kinase cyclin-dependent kinase 9 (CDK9) and its regulatory subunit cyclin L1 in prostate cancer cells [32]. The formation of this trimeric ribonucleoprotein complex promotes the phosphorylation of RNAPII CTD serine-2 residues, which stimulates promoter escape of RNAPII and productive elongation at the Runx family transcription factor 2 (RUNX2) promoter. These findings suggest that NEAT1 transcripts control the production and processing of a subset of protein-coding transcripts both within paraspeckles and on chromatin. However, it is currently not well understood if the chromatin-associated functions of NEAT1 transcripts are isoform specific. NEAT1 isoforms modulate gene expression by tethering RBPs and transcripts to paraspeckles, but also scaffold the formation of regulatory ribonucleoprotein complexes on chromatin and in the cytoplasm. The versatility in NEAT1-associated RBPs and transcripts underscores the important need to develop novel methods to dissect the NEAT1 interactome.

## 3. An Ever-Growing Toolbox for the Assessment of Ribonucleoprotein Complexes

Recent technological developments have advanced our understanding of RNA-protein and RNA-RNA interactions. RNA interactome studies are often based on the isolation of native or crosslinked complexes, which are subjected to mass spectrometry (MS) and next generation sequencing (NGS) to identify the proteins and transcripts that interact or associate with the bait RNA. New technology like Protein-crosslinked RNA extraction (XRNAX), RNA-dependent protein ultracentrifugation (R-deep) and RNA in situ conformation sequencing (RIC-seq) allows the unbiased identification of novel RBPs and their associated transcripts, as well as RNA-RNA interaction mapping at a global scale [57,58,59]. At the level of individual transcripts many biochemical methods to assess the RNA interactome are based on high affinity to, and selective enrichment of, the RNA of interest [15]. Such affinity can, for example, be provided by in vitro-transcribed complementary RNA baits. The in vitro transcript of interest is immobilised on beads and used as a bait in pull-down assays with whole cell lysates. This method is favoured to assess the interactome of shorter transcripts of <100 nts such as sncRNA [60]. The identification of RBPs that associate with lncRNA often requires more advanced biochemical methods like RNA affinity purification (RAP). In addition, the two RAP variants Capture hybridisation analysis of RNA targets (CHART) and Chomatin isolation by RNA purification (ChiRP) also assess the interactions of lncRNA with chromatin [61,62,63,64,65]. These methods utilise modified DNA antisense oligonucleotides, which hybridise with the complementary endogenous RNA of interest. The DNA probes are very stable and often end-modified with high affinity tags such as biotin or digoxigenin, which allows for stringent immunoselection of captured ribonucleoprotein complexes. However, a large panel of overlapping DNA probes may be required for efficient capture of less abundant transcripts. Some probes may also compete with endogenous RBPs for binding, alter the secondary structure of the RNA of interest, or cross react with collateral transcripts, which demands effective controls such as scrambled probe sets. Some off-target effects can, for instance, be reduced by ribonuclease H (RNaseH)-mapping, which determines the regions within the RNA of interest that are accessible to the capturing DNA probes. 

The biochemical enrichment of endogenous ribonucleoprotein complexes by pull-down assays and complementary DNA probes is performed in vitro and requires cell lysis. As an alternative, RNA aptamer systems can be used in vivo, without the need to interfere with subcellular compartmentalisation. However, the usage of aptamers requires genetic manipulation. Either the endogenous locus, which encodes the RNA of interest, can be tagged with aptamer sequences, or a recombinant fusion gene can be cloned and ectopically expressed. Although various types of aptamers exist, the MS2 system is most widely used. MS2 aptamers contain short RNA sequences that form an array of stem loops, which are recognised by specific MS2-binding proteins (MBPs) such as the MS2 coat protein (MCP). The coexpression and immunoselection of an affinity-tagged MBP allow the purification of MS2-associated ribonucleoprotein complexes. The MS2 system is highly versatile and has been applied for a wide range of RNA species of different lengths [66,67,68]. Nevertheless, the expression of endogenously tagged, or ectopically expressed, MS2 fusion transcripts may alter the accessibility of endogenous RBPs to the RNA of interest and may impact on the correct formation of the secondary structures and the stability of the fusion transcript.

Interestingly, many of the described strategies to capture RNA interactomes have been applied for NEAT1. The biochemical purification of NEAT1-associated proteins by RAP and CHART gleaned novel insights into the regulation of NEAT1 transcription, paraspeckles assembly and NEAT1-mediated promoter regulation on chromatin. As discussed above, RAP revealed several components of the Integrator complex as newly identified interactors of NEAT1 in MCF-7 cells [30]. In total, the RAP approach identified 32 proteins that associate with NEAT1 with high confidence. About 50% of them are known core paraspeckle components. The association of many of the 32 candidates could be confirmed by immunoblotting and enhanced crosslinking by UV and immunoprecipitation, followed by high-throughput sequencing (eCLIP-seq). Remarkably, the composition of the RAP-derived NEAT1 interactome was largely insensitive to the induction NEAT1 synthesis by DNA damage or oncogenic stress. Unlike RAP, the assessment of the NEAT1 interactome by CHART allows the additional mapping of NEAT1 associations with chromatin [52]. Combining the enrichment of NEAT1 by CHART with deep sequencing, West and colleagues could show that NEAT1 localises to hundreds of actively transcribed protein-coding gene loci in MCF-7 cells. The accumulation of NEAT1 on chromatin is particularly strong at transcription start sites and transcription exit sites, and comprises significant overlay with the metastasis-associated lung adenocarcinoma transcript 1 (MALAT1). Moreover, the accumulation of NEAT1 on protein-coding gene promoters occurs in trans, is sensitive to inhibitors of RNAPII transcription, and is accompanied by several newly identified NEAT1 interactors.

The genomic tagging of the NEAT1 locus with MS2 stem loops and the transient expression of MS2-NEAT1_1 constructs allow the enrichment of NEAT1-associated RBPs in vivo. The immunoselection of MS2 ribonucleoprotein complexes revealed that the ELAV-like RNA-binding protein 1 (ELAVL1/HuR) binds NEAT1 to promote NEAT1 transcript stability [69]. Recently, a global analysis of the NEAT1 interactome was performed by using the CRISPR-mediated endogenous lncRNA tracking and immunoprecipitation system (CERTIS) [70]. For CERTIS, an array of 24 MS2-tag repeats was inserted in the distal end of the NEAT1 locus of HEK293T cells. CERTIS allows both the tracking of NEAT1 transcripts in living cells and the isolation of NEAT1-associated RBPs by immunoselection. Indeed, the combination of CERTIS with mass spectrometry yielded a list of 174 putative NEAT1-associated RBPs, which includes many known paraspeckle components, but also the novel NEAT1 interactor quaking (QKI). Importantly, the MS2-tag did not interfere with the expression level, distribution or function of NEAT1 in HEK293T cells. 

Thus, a growing number of NEAT1 interactome studies reveal both mechanistic insights in the synthesis of paraspeckle-seeding NEAT1 transcripts and suggest a role for chromatin-associated NEAT1 in the regulation of RNAPII transcription. However, the discussed approaches are often laborious, require substantial amounts of starting material, and may not always be readily applicable for the cellular system of interest. In fact, they may be less suitable for the mapping of lncRNA-associated proteins and transcripts at compartment-wide scale, as it is the case for NEAT1 and paraspeckles. 

## 4. Assessment of Ribonucleoprotein Complexes by Proximity Ligation

Proximity ligation is a powerful strategy to address some of these obstacles [71]. Proximity ligation is based on promiscuous non-mammalian enzymes such as the engineered ascorbate peroxidase 2 (APEX2) or the biotin ligase BirA*, which can be expressed in mammalian cells and targeted to specific subcellular localisations or compartments to biotinylate proximal proteins and transcripts in vivo [68,72,73,74,75,76,77]. Proximity ligation approaches gained important insights into the organisation of subcellular compartments and the RNA repertoire of nuclear bodies such as stress granules [78,79]. Mechanistically, APEX2 uses hydrogen peroxide (H_2_O_2_) and biotin-phenol to generate highly reactive and short-lived biotin radicals that conjugate with proximal endogenous amino acids and nucleic acids [80]. APEX2-derived biotin radicals are produced with enhanced reaction kinetics, do not penetrate across membranes and have a favourable labeling radius compared to BirA*. However, the efficacy of substrate biotinylation can be influenced by the local cellular environment such as heterogenic concentrations of glutathione and discontinuous protein density [81]. Further, the compartment-specific biotinylation by APEX2 requires cloning of an APEX2 fusion protein and is limited to subcellular compartments that are amenable to trafficking signals in vivo. The stable expression and bona fide localisation of such APEX2 fusion constructs can be challenging to achieve, particularly in difficult to transfect systems like primary cells or clinical samples. Recombinantly expressed APEX2 fusion proteins must also be monitored for activity and cytotoxicity to avoid non-physiological results, reflecting the need for additional proximity ligation methods.

## 5. The Hybridisation Proximity Technology

**Figure 2:**
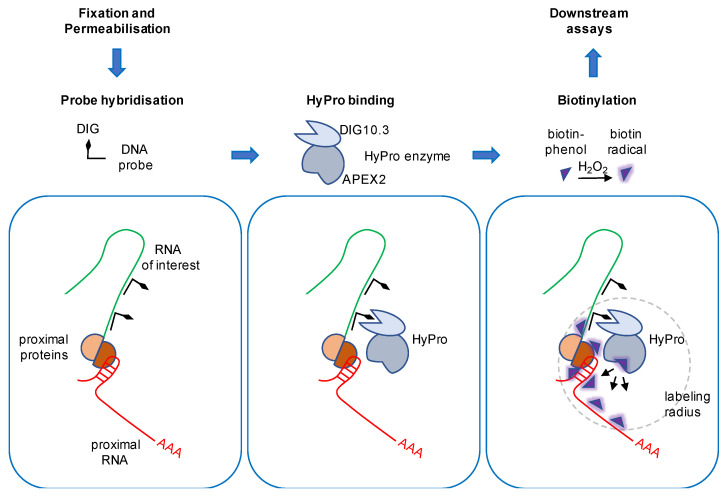
The HyPro labeling workflow. HyPro labeling is performed in mammalian cells of interest. For probe hybridisation, a panel of digoxigenin (DIG)-labeled DNA oligonucleotides (black) that are complementary to the RNA of interest (green) are added to fixed and permeabilised cells. Next, the purified HyPro enzyme is added. The recombinant fusion protein consists of the engineered digoxigenin-binding protein DIG10.3 and the engineered ascorbate peroxidase 2 (APEX2). High affinity binding of DIG10.3 to DIG tethers HyPro to the RNA of interest. For the biotinylation of proximal proteins and transcripts (red), biotin-phenol (purple) is added and converted to reactive biotin-radicals (purple glow) in the presence of hydrogen peroxide (H_2_O_2_) for a few minutes. Proximity ligation occurs within a labeling radius of 20 nm (grey). Subsequently, cells are lysed, biotinylated molecules are purified and subjected to downstream assays such as mass spectrometry and deep sequencing. Schematic illustrations were adopted from Yap and colleagues [17,82].

HyPro labeling has recently significantly advanced the spectrum of APEX2-based omics approaches [17]. This in situ variant of proximity labeling is able to profile RNA-protein and RNA-RNA proximity patterns in genetically unmodified samples (Figure 2). Proximity ligation is performed by the HyPro enzyme, a custom-designed, bifunctional fusion protein that consists of a codon-optimised bacterial APEX2 version and the engineered digoxigenin-binding protein DIG10.3. The method utilises digoxigenin-labeled DNA antisense probes that selectively recognise lncRNA transcripts of interest by complementary hybridisation in fixed cells. Prior to hybridisation, cells are crosslinked with the Lomant’s reagent DSP and permeabilised with ethanol. For labeling, the fixed material is incubated with the recombinant purified HyPro enzyme. The DIG10.3 domain of HyPro binds the digoxigenin-labeled DNA-RNA hybrids with subnanomolar affinity, whilst non-bound HyPro is washed off. This allows the specific biotinylation of immobilised proteins and transcripts in close proximity to the RNA of interest upon addition of biotin-phenol and H_2_O_2_. Subsequently, the ligation reaction is quenched, cells are lysed and crosslinks are reversed. The labeled proteins and transcripts are captured in denaturing conditions by streptavidin-coated magnetic beads, eluted and subjected to downstream assays. Importantly, the HyPro fusion protein weighs less than 50 kDa, which favours high accessibility to crowded molecular environments and restrains the labeling radius to approximately 20 nm. The HyPro technology was initially established in HeLa cells and its applicability was confirmed in non-transformed APRE-19 cells, as well as primary stem cells. To demonstrate that the hybridisation is specific and the fusion protein catalytically active, Yap and colleagues tested the activity of the HyPro enzyme first in vitro, using assays to measure the peroxidase activity and digoxigenin binding, and then in situ, using confocal microscopy. The HyPro enzyme was targeted to three lncRNA transcripts that seed three distinct compartments: (i) the highly abundant nucleolar 45S rRNA, (ii) the paraspeckles-assembling lncRNA NEAT1, and (iii) the pyrimidine-rich non-coding transcript (PNCTR), a lower expressed lncRNA that marks the PNC. Combining fluorescently labeled streptavidin with antibodies against compartment-specific marker proteins, the authors could show that the streptavidin signals specifically colocalised with the targeted compartments upon addition of the APEX2 substrates biotin-phenol and H_2_O_2_. In contrast, the streptavidin signals were observed throughout the cell when biotinylation was performed with infused HyPro in absence of DNA probes. No signals were observed when scrambled probes were used. The colocalisation with compartment markers was also observed with a digoxigenin antibody after DNA probes were hybridised and no HyPro enzyme was added. Thus, the HyPro enzyme can selectively be targeted to three structural lncRNA transcripts that are expressed in different compartments and at different levels. 

## 6. HyPro Labeling Expands the NEAT1 Interactome

**Figure 3:**
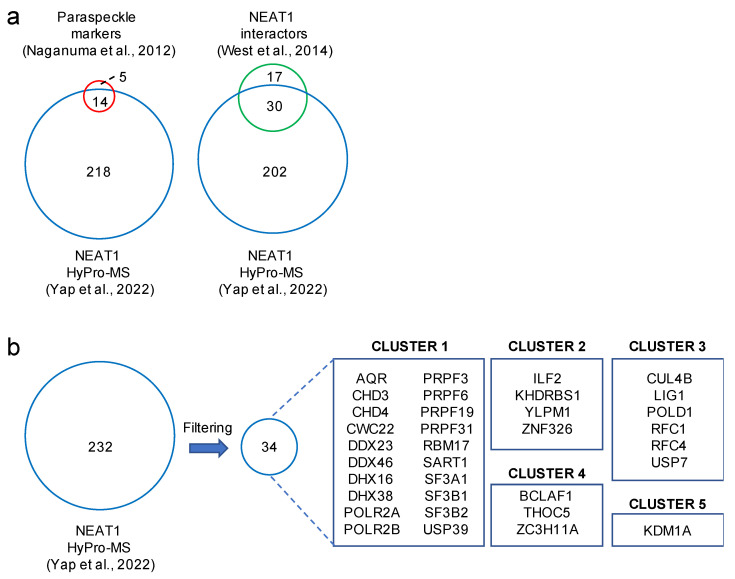
HyPro-mass spectrometry (HyPro-MS) identifies novel NEAT1 interactors. (**a**) Venn diagrams display significant overlap of 232 NEAT1 HyPro-MS candidates (light blue, [17]) with 19 paraspeckle markers previously identified by confocal imaging (light red, [26]) and 47 NEAT1 interactors previously identified by mass spectrometry (light green, [52]). (**b**) Filtering of NEAT1 HyPro-MS candidates for high-confidence protein sets, exclusion of candidates from cell biological imaging [26] and biochemical purification [52], molecular complex detection software, and gene ontology analysis results in 5 clusters of 34 newly identified NEAT1 interaction candidates. The 5 clusters were defined by Yap and colleagues as described, but do contain additional known paraspeckles components, which are not depicted here.

HyPro labeling can be combined with MS and NGS to glean insights into RNA interactomes. When applied for NEAT1, MS data contained 232 proteins that were significantly enriched over the background (Figure 3a). Strikingly, the NEAT1-associated proteome contained 14 out of 19 core paraspeckle components, including NONO and SFPQ [26]. Further, HyPro labeling confirmed 30 out of 47 previously described NEAT1 interactors [52]. HyPro labeling yielded 202 additional candidates. A total of 34 highly significant novel hits were identified (Figure 3b). The bulk of the hits clustered into gene ontologies that demarcate RNA metabolism and chromatin organisation. Reassuringly, the composition of the NEAT1-specific proteome was substantially different compared to the 45S rRNA-specific proteome, albeit some paraspeckles tend to form in the vicinity of nucleoli [21]. 

The authors also characterised compartment-specific transcriptomes by combining HyPro labeling with NGS. Profiling for NEAT1-associated transcripts revealed a set of 267 transcripts that was significantly distinct compared to nucleolar transcripts. Besides enrichment of the NEAT1 bait itself, an enhanced proportion of coding transcripts (e.g., NAA40, CCDC57, PC; EML3) and a subset of miRNA precursors (e.g., MIR17, MIR24-2, MIR27A) were prominently biotinylated in the NEAT1-specific HyPro labeling. The association of some mRNA with paraspeckles was validated by RNA fluorescence in situ hybridisation (RNA-FISH) stainings, which confirmed the function of paraspeckles in mRNA retention and miRNA biogenesis. A more detailed bioinformatic analysis revealed that the genes that encode the set of HyPro-labeled, NEAT1-associated transcripts are arranged on the genome in a highly non-uniform manner [17]. Indeed, the bulk of NEAT1-associated transcripts originates from a 16 Mb region on the q-arm of chr11, which contains the NEAT1 locus itself and telomere-proximal parts of chr11p, chr9q, and chr17q. Yap and colleagues could demonstrate that the origin of NEAT1-specific HyPro labeled transcripts shows little overlap with 45S rRNA-specific labeled transcripts, which were mostly mapped to the nucleolar organising region-containing chromosomes 15, 21, and 22. In addition, HyPro labeling revealed that NEAT1-associated transcripts are often incompletely processed and A-to-I edited. The NEAT1-associated mRNA transcripts NAA40 and CCDC57, for instance, comprise a strong retention of introns and accumulation of RNA sequencing reads at the 3′ end, indicating enhanced read-through of RNAPII. Indeed, the depletion of NEAT1 both diminished the formation of paraspeckles and reduced the level of HyPro-labeled transcripts. Likewise, co-staining of NEAT1 with NAA40 or CCDC57 was strongly diminished upon the depletion of NEAT1 in RNA-FISH experiments. Thus, HyPro labeling identifies novel RBPs and transcripts that selectively associate with paraspeckles.

## 7. Limitations and Improvements of HyPro Labeling

HyPro labeling was successfully performed for both abundant 45S rRNA and NEAT1, as well as for PNCTR, a lncRNA expressed at less than 50 copies per cell. Albeit a protocol with highly efficient conditions for crosslinking, permeabilisation, hybridisation and labeling has been described [82], additional optimisation, in terms of specificity and sensitivity (e.g. increased number of cells or prolonged labeling time) may be required to investigate lowly expressed RNA baits. 

A critical limitation of APEX2-mediated biotinylation is the relatively large labeling radius, which potentially increases the number of false-positive candidates for some smaller RNA compartments [17]. As performed by Yap and colleagues, cross validation of candidate lists with published data sets can reduce the number of false-positive hits. As a complementary approach, the interactome of individual transcripts can also be assessed by APEX2 ligases that utilise the CRISPR/dCas13 system. Indeed, dCas13-guided proximity ligation gained novel insights into the interactomes of other lncRNA such as MALAT1 and the non-coding RNA activated by DNA damage (NORAD) in HEK293T cells [83,84], and could be adopted for NEAT1 for cross validation. However, dCas13-mediated RNA-targeting and proximity ligation require the expression and guide RNA-mediated targeting of a catalytically inactive dCas13-APEX2 fusion protein in vivo. The relatively large size of dCas13 may also compete for binding with cognate RBPs and the guidance of dCas13 to the lncRNA of interest may be biased for regions that are not fully occupied by RBPs. Alternatively, other proximity labeling enzymes (e.g. the proteasomal accessory factor A (PafA) ligase, or a split-APEX2 variant) may be fused to DIG10.3 to either reduce the labeling radius or enable specific, diffusion-less proximity ligation of substrates [73,85,86]. 

Furthermore, the design of the targeting DNA probes requires attention. Both specificity and accessibility issues might arise, especially when targeting lowly expressed RNA baits or structural transcripts that are heavily coated with RBPs. A subset of DNA probes likely competes with endogenous RBPs for transcript binding and results in an underestimation of associated proteins [17,82]. Thus, the usage of more than one probe set is recommended, along with methods like RNA-FISH and RNaseH protection assays to validate probe sets. As discussed by Yap and colleagues, an increase in the amount of DNA probes or the substitution of biotin-phenol with highly favourable APEX2 substrates may further enhance HyPro sensitivity and specificity. Biotin-aniline and biotin-naphthylamine, for example, are novel probes that comprise substantially higher affinity to nucleic acids compared to biotin-phenol [87]. Of note, the DNA probe set used by Yap and colleagues to target NEAT1 did not discriminate between NEAT1_1 and NEAT1_2 isoforms. It remains to be established to what extent the differential targeting of the HyPro enzyme to individual NEAT1 isoforms is technically feasible, as the sequence of NEAT1_1 is identical to NEAT1_2 and no cytoplasmic lncRNA has yet been assessed by HyPro labeling. 

Interestingly, genome engineering of the NEAT1 locus allows the modulation of the ratio of NEAT1 isoforms. In particular, the destruction of the polyadenylation site at the 3′ end of NEAT1_1 by CRISPR/Cas9 editing diminishes the NEAT1_1 level and favours the synthesis of NEAT1_2. Given that NEAT1_1 and NEAT1_2 regulate gene expression at various levels, it will be important to combine genome engineering of the NEAT1 locus with yet to be developed variants of HyPro labeling to potentially gain insights into NEAT1 isoform-specific interactomes in different compartments.

## 8. Clinical Perspectives

HyPro labeling identifies dynamic changes in RBPs and transcripts associated with NEAT1 and, thus, may be a powerful approach to assess the function of paraspeckles in the context of diseases such as cancer. Interestingly, RNAPII transcription at the NEAT1 promoter is responsive to various kinds of stress. NEAT1 is a direct target of the heat shock transcription factor 1 (HSF1), the hypoxia-inducible factor 2 alpha (EPAS1/HIF2A) and the activating transcription factor 2 (ATF2). These transcription factors upregulate NEAT1 expression in various human tissue culture cells in response to heat shock, hypoxic conditions and mitochondrial stress, respectively [53,88,89]. These findings suggest that an increase in the number or in the size of paraspeckles mitigates cellular stress, which indicates that the composition of paraspeckles-associated factors is responsive to stimuli. Interestingly, NEAT1 is upregulated in about 65% of tumours, including breast cancer, prostate cancer, colon cancer, lung cancer, ovarian cancer, and pancreatic cancer [44,90,91,92]. Elevated levels of NEAT1 isoforms generally promote tumour development in mouse models and function as a prognostic marker for patient survival [93]. In colon cancer, for example, high levels of NEAT1 correlate with enhanced growth and proliferation, as well as poor prognosis for disease-free survival [49]. On the other hand, NEAT1 transcripts can also act as a tumour suppressor. In AML, for instance, low levels of NEAT1_1 correlate with enhanced relapse of the tumour and de-repression of Wnt signaling [48]. NEAT1_2 expression is also sensitive to oncogenic stress. The tumour suppressor protein p53 (TP53) upregulates NEAT1_2 to prevent transformation and replication of stress-induced DNA damage in mammalian cells [94]. Thus, the induction of NEAT1_2 is regarded as tumour suppressive, whereas high levels of NEAT1_1 have oncogenic consequences, suggesting that NEAT1 may have a dual role in cancer formation. However, it should be mentioned that in quite a few cases the reported changes in NEAT1 level do not distinguish between NEAT1 isoforms.

What are the molecular mechanisms that help to distinguish NEAT1 isoforms during oncogenic transformation or in response to stress? Is the metabolism of NEAT1 transcripts druggable and could it potentially be exploited to interfere with cancer-associated NEAT1 functions? Arguably, the expression levels and the ratio of NEAT1 isoforms in tumours are regulated by a complex interplay of oncogenes and tumour suppressors that steer the transcription, 3′ end formation, localisation and stability of NEAT1 isoforms. Oncogenes like the octamer-binding transcription factor 4 (POU5F1/OCT4) and the epidermal growth factor (EGF) induce the expression of both NEAT1 isoforms, whilst c-Myc seems to be a negative regulator of NEAT1 levels [90]. The deregulated NEAT1 RNA metabolism of cancer cells, in turn, may alter gene expression in tumours at various levels: in the context of paraspeckles, on chromatin and in the cytoplasm. It is currently not well understood which steps in the metabolism of NEAT1 are deregulated and may be critical for oncogenic transformation. One possibility is that changes in the NEAT1 isoform ratio have tumour- or tissue-specific effects. Some cancers may require high levels of NEAT1_2 to form tumour-specific associations of NEAT1_2 with RBPs in the context of paraspeckles. NEAT1_2 may be required for the control of growth and proliferation, or perhaps limit the availability of RBPs that restrain transformation by sequestration to paraspeckles. On the other hand, the newly described roles of NEAT1_1 in the cytoplasm may also be relevant for tumourigenesis. High levels of NEAT1_1 might stimulate an enhanced metabolic energy flux that is fostering metastasis. In both scenarios, HyPro labeling is a technology of choice to understand the highly dynamic changes in lncRNA-associated interactomes. The application of HyPro labeling in patient samples promises to be a powerful tool to understand how NEAT1 could modulate gene expression in tumours and may pave the way for the identification of novel tumour targets. Current approaches to interfere with NEAT1 function in cancer cells include the application of antisense oligonucleotides (ASOs) that impair NEAT1 synthesis [95]. The application of ASOs that target the NEAT1 isoform switch region sterically block NEAT1_1 polyadenylation and trigger the loss of NEAT1_1 transcripts concomitant with high levels of the NEAT1_2 isoform, and have a tumour-suppressive effect in neuroblastoma cells. The non-genotoxic ASO strategy to block NEAT1_1 could be combined with the inhibition of druggable paraspeckle components that hyper-activate the RNA metabolism in tumours—presumably identified by HyPro labeling—and may be of therapeutic benefit in the future. 

We have only just begun to understand the mechanistic principles that link the production and processing of NEAT1 transcripts with paraspeckles biology and tumourigenesis. Novel methods such as HyPro labeling may help to close the technological gap that currently limits our understanding of the likely widespread and underestimated regulatory roles of the non-coding genome in the regulation of gene expression and tumourigenesis. 

## Data Availability

Not applicable.
